# Permanent areas and changes in forests, grasslands, and wetlands in the North European Plain since the eighteenth century—a case study of the Kościan Plain in Poland

**DOI:** 10.1038/s41598-024-61086-3

**Published:** 2024-05-05

**Authors:** Beata Medyńska-Gulij, Krzysztof Szoszkiewicz, Paweł Cybulski, Łukasz Wielebski

**Affiliations:** 1 Department of Cartography and Geomatics, Adam Mickiewicz University, Poznań, Krygowskiego 10, 61-680 Poznan, Poland; 2 Department of Ecology and Environmental Protection, Poznań University of Life Sciences, Poznan, Poland

**Keywords:** Land cover changes, Topographic map, Grasslands, Forests, Wetlands, North European Plain, Forest ecology, Grassland ecology, Wetlands ecology, Sustainability

## Abstract

This study investigates the intricate and enduring interplay of historical events, human activities, and natural processes shaping the landscape of North European Plain in western Poland over 230 years. Topographic maps serve as reliable historical data sources to quantify changes in forest, grassland, and wetland areas, scrutinizing their fragmentation and persistence. The primary objectives are to identify the permanent areas of the landscape and propose a universal cartographic visualization method for effectively mapping these changes. Using topographic maps and historical data, this research quantifies land cover changes, especially in forest, grassland, and wetland areas. With the help of retrogressive method we process raster historical data into vector-based information. Over time, wetlands experienced a substantial reduction, particularly in 1960–1982, attributed to both land reclamation and environmental factors. Grassland areas fluctuated, influenced by wetland and drier habitat dynamics. Fragmentation in grassland areas poses biodiversity and ecosystem health concerns, whereas forested areas showed limited fluctuations, with wetland forests nearly disappearing. These findings highlight wetland ecosystems’ sensitivity to human impacts and emphasize the need to balance conservation and sustainable development to preserve ecological integrity. This study advances landscape dynamics understanding, providing insights into historical, demographic, economic, and environmental transformations. It underscores the imperative for sustainable land management and conservation efforts to mitigate human impacts on ecosystems and biodiversity in the North European Plain.

## Introduction

Land cover plays a significant role in the environment and human life, determining the landscape and character of an area, the species of plants and animals inhabiting a given territory, as well as the economic^1,2^ and touristic^3,4^ potential of the lands. Human activity, along with the development of settlements, agriculture, and later industry, has led to transformations in the natural landscape^5,6^. The intensification of this intervention is also facilitated by population growth and technological advancement. Humans alter the landscape to accommodate their needs, and with the advancement of knowledge, this can be done in a more responsible and sustainable manner.

It is widely accepted that the analysis of alterations in land cover since the pre-industrial era could reveal pivotal events in Land Use and Land Cover (LULC) changes, particularly evident in the context of long-term change studies derived from topographical sources^7,8^. The analysis of LULC changes holds significance as it serves as a vital indicator required for the realization of the United Nations' Sustainable Development Goals (SDGs)^9–11^. LULC change studies based on topographic materials often focus on several elements such as grasslands, forests, urban areas, or arable land^12–14^.

In the context of Forest Transition Theory^15,16^, the forest lands in the European Plains were cleared for agricultural purposes^17,18^, while the remaining areas were heavily managed for timber production^19,20^. As a result, many countries in the region have implemented policies and programs aimed at promoting sustainable forest management practices, conserving biodiversity, and protecting forest ecosystems^21,22^, which might be recognized as a reversal of the deforestation trend^23^.

To track changes in Land Use and Land Cover (LULC) and incorporate them into a specific theoretical trend, methods based on the analysis of topographic maps are employed, as observed in the case of mountain landscapes^24–28^. Some research has focused on parts of the European Plain^29–32^ incorporating grasslands that have consequently disappeared and fragmented due to agricultural development^33,34^.

In recent years, the drainage of wetlands for agriculture has slowed down in Europe, but the wetlands continue to face threats from urbanization and other forms of development^35,36^ as well as climate change^37^. However, it is rare to find scientific research that examines the wetlands, their changes, and stability in Greater Poland based on topographic materials from the eighteenth century^38,39^. These processes might lead to rapid and abrupt changes, resulting in a regime shift that limits the possibility of predicting future changes^40,41^.

Therefore, the question arises: Have there been such significant changes in the area of forests, meadows, and wetlands over the past 230 years on the Kościan Plain that their continuity has been interrupted?

Cartographic visualizations depicting changes in land cover can assume two distinct approaches^42^. The first option entails an analytical approach, where the presentation method and data granularity allow for a detailed investigation of changes. Such visualizations can be challenging to interpret, and their users are typically experts who utilize them as tools for studying and understanding the phenomenon^43^. In such cases, geographic visualization (geovisualization) is often employed, involving interactive tools to enhance analysis efficiency^44–47^. The second approach involves simplifying the data, making the information more easily communicable in a graphical manner, thereby reaching a broader audience.

Another research question that arises is: How can cartographic visualization reveal permanent landscape features since the eighteenth century using topographic maps?

The goal of this study is to assess changes in forest, grassland, and wetland area and recognizing permanent areas in the Kościan Plain in Poland on the European Plain over the past 230 years on a topographical scale, using topographic maps. Specifically, we aim to assess changes in forest, grassland, and wetland area, their fragmentation, and durability. We also propose a universal method for cartographic visualization to support the visualization of changes in forest, grassland, and wetland areas in the North European Plain.

The main research hypothesis posits that the existence of permanent areas within grasslands and forests on the Kościan Plain has played a crucial role in preserving these landscapes over the past 230 years. These permanent areas, coupled with the conservation of drainage canals, have been instrumental in sustaining substantial portions of permanent grasslands and forests despite landscape alterations. The second research hypothesis is that the cartographic method proposed in a local study will allow determining the location and area of forests, grasslands and wetlands that have a permanent character. Unlike other studies of this type that have been mentioned here, we would also like to include wetlands in the landscape analysis. Wetlands are not a common element of analysis based on cartographic materials. Moreover, we aim to supplement this type of research with a universal method of cartographic presentation that can be employed regardless of the type of landscape.

## Materials and methods

### Study area

**Figure 1:**
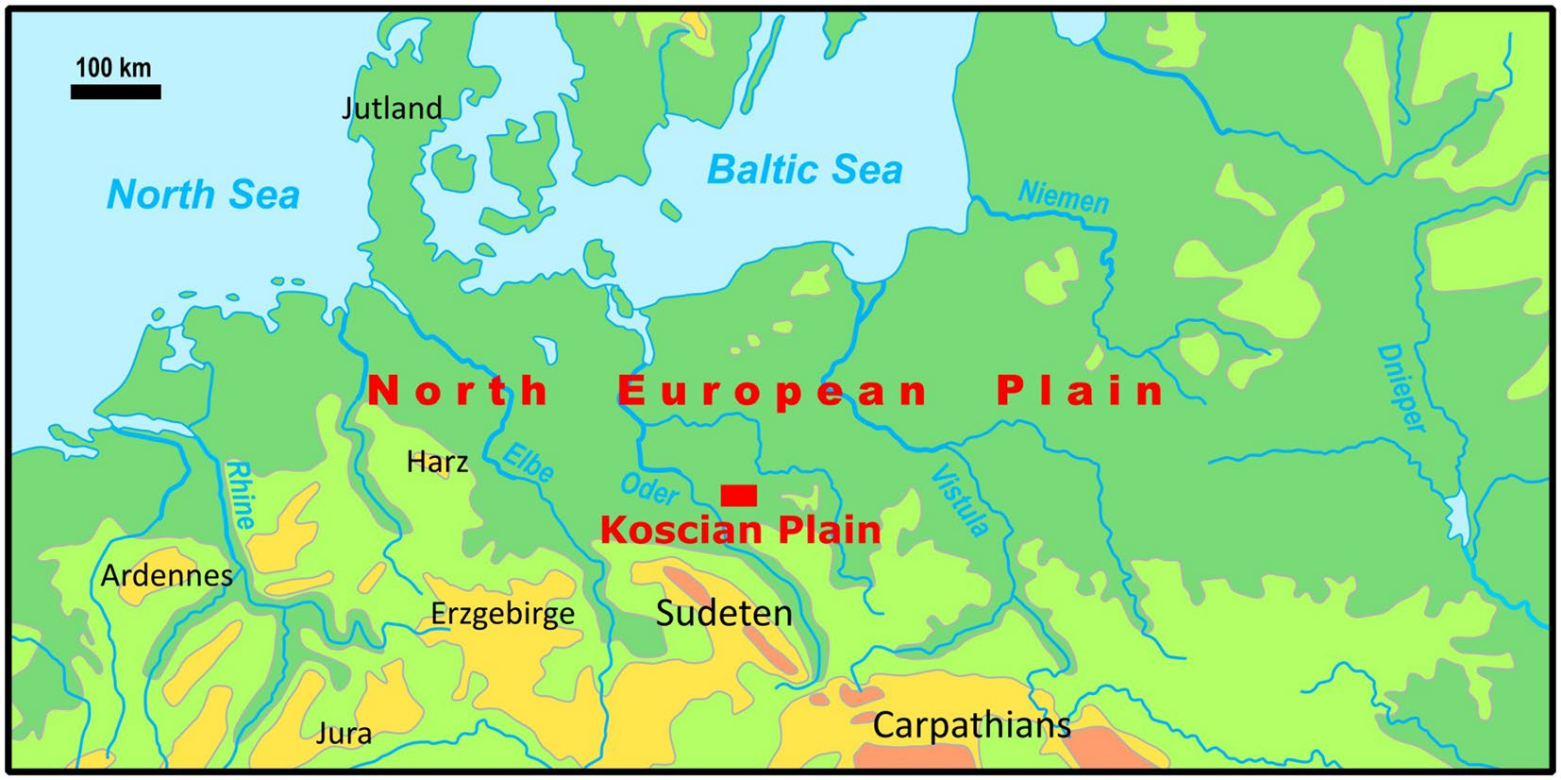
Localization of the Kościan Plain on the North European Plain.

The Kościan Plain is a significant geographic region within the context of the North European Plain, located in western Poland (Greater Poland, Wielkopolska), covering an area of 194 km2, as illustrated in Fig. 1. It is distinguished by its relatively flat topography, fertile soils, and a mosaic of land cover types, including forests, grasslands, and wetlands. This region plays a crucial role in the broader North European Plain, extending from the southern coast of the Baltic Sea to the Alpine foothills in the south.

Representativeness of the Kościan Plain for the North European Plain in the context of cartographic materials results from access to credible and continuous cartographic material (including the first professional topographic maps created in the eighteenth century) compared to similar consistent materials, e.g., Lower Saxony, Brandenburg, the Netherlands, Greater Poland, and Mazovia^48–51^. The territorial limitation of our research to the shape and size of map sheet and regular sheet division for topographic map aimed to propose a universal methodological approach to each fragment of the North European Plain, as its elementary part.

Forests have been an essential feature of the Kościan Plain’s landscape, contributing to its ecological diversity and providing numerous ecosystem services. The changes in land cover in the Greater Poland (Wielkopolska) were documented the impact of deforestation for agricultural expansion and urban development^39^. Additionally, the effects of afforestation efforts on restoring forest ecosystems and their implications for the spread of the pollutions in ground waters and in soils were investigated^52^.

Due to changes in land cover, some grasslands, which have a permanent character^53^, not only reduce their surface area, but also their functionality^54^. Historically, grasslands have been an important part of Poland’s landscape, providing habitat for a wide variety of plant and animal species^55,56^. However, in recent years, many grasslands have been lost or degraded due to changes in land cover, such as the conversion of grasslands to agriculture or urban areas, and the abandonment of traditional land management practices^57^. In addition, the loss of grasslands can also have negative impacts on soil quality, water retention, and carbon storage^58,59^. To address these challenges, efforts are underway in Poland to protect and restore grasslands^60^. Grasslands have also been a crucial component of the Kościan Plain’s land cover. Traditionally used for sustainable agriculture and livestock grazing, grasslands have faced significant transformations due to agricultural intensification and changes in land management practices^61^. They also had the flood protection function. Research by Kozaczyk^62^ examined the water regulation impact on the permanent grasslands and their exploitation.

Historically, large areas of wetlands were drained for agricultural purposes, particularly in the Netherlands, Germany from the sixteenth century, and later in also Poland^63^. In western Poland, land reclamation was carried out on a small scale in the nineteenth century and on a much larger scale in the twentieth century, especially in the 1960s and by the early 1980s^62,64^. Wetlands, including marshes and peatlands, were once abundant in the Kościan Plain, contributing to water regulation, carbon sequestration, and wildlife habitat. However, Mizgajski^65^ highlighted the alarming decline of wetlands in the region due to drainage and land reclamation for agricultural purposes, especially in the nineteenth century. This has raised concerns about the loss of valuable wetland ecosystems and the potential implications for flood control and water quality.

Understanding the changes in forest, grassland, and wetland cover in the Kościan Plain is essential in the context of broader European Plains. It allows us to assess the impacts of land cover changes on regional biodiversity, ecosystem services, and environmental sustainability. The integration of this regional knowledge into broader European studies, such as those conducted by Wulf and Gross^66^ on pan-European land cover changes, enables a comprehensive understanding of the complex interactions between human activities and the natural environment across the continent.

### Data

For research on land cover in the European Plain for Western Poland, there is high potential but still unexplored.

Manuscript topographical maps from the second half of the eighteenth century constitute a unique documentation of pre-industrial space in Europe. This uniqueness results not only from the fact that these are the first cartographic records occurring in a single copy, but also from the fact that they are based on direct field observations. Field mapping was executed through table sketches using surveying tools and observations^49^. Due to their specific cartometric nature and interpretational possibilities, these works have been the subject of few studies until now^48,50,67^.

Table 1 presents a set of source maps with the following information: map name, publication year/years, scale, author/institution, provenance/owner, and publication technique. The year associated with each map in Table 1 indicates the approximate year in which forest, meadow, and wetland areas were mapped.

**Table 1:** Cartographic sources by provenance.

Year		Title of topographic map	Scale	RMSE [m]	Publishing techniques
1793	**A**	Karte von Südpreußen (Map of Southern Prussia)	Ca. 1:50,000	83	Manuscript map
1826	**B**	Urmesstischblätter	1:25,000	75	Manuscript map
1892	**C**	Messtischblätter I	1:25,000	20	Printed map
1944	**D**	Messtischblätter II	1:25,000	19	Printed map
1960	**E**	Powiatówka (County map)	1:25,000	31	Printed map
1982	**F**	Topographic map „1965”	1:50,000	0,09	Printed map
1998	**G**	Topographic map „1992”	1:50,000	0,05	Printed map
2023	**H**	BDOT10k (Database of Topographic Objects)	1:10,000 (1:50,000)	_	Vector map

The Berlin State Library in Germany: A, Karte von Südpreußen (Map of Southern Prussia); B, Urmesstischblätter; C Messtischblätter I; D, Messtischblätter II; The Head Office of Geodesy and Cartography in Poland: E, Powiatówka (County map); F, Topographic map “1965”; G, Topographic map “1992”; H, BDOT (Database of Topographic Objects).

**Figure 2:**
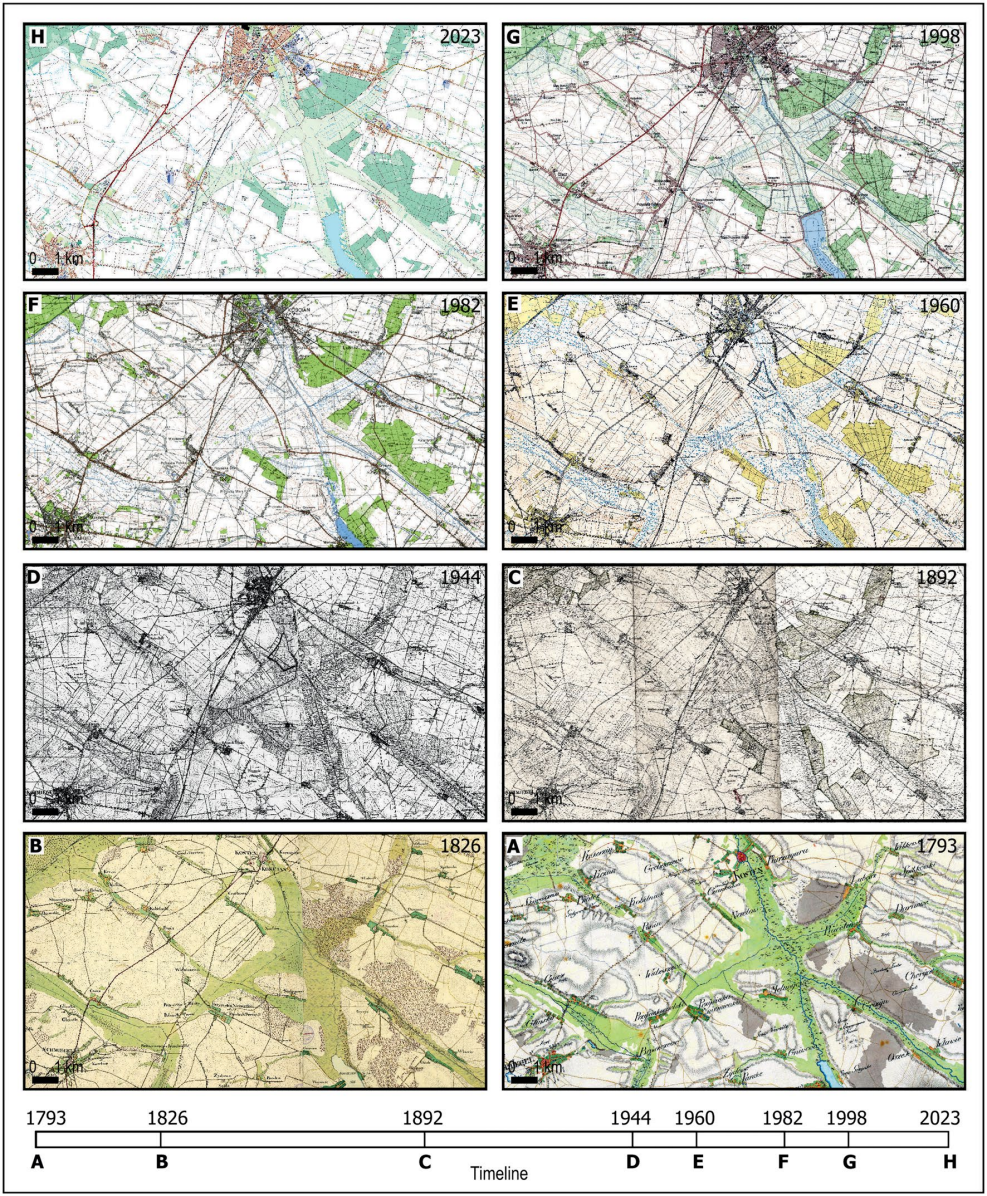
Topographic maps of Kościan Plain from 1793 to 2023, according to the description in Table 1. An illustrative timeline is provided below the maps, aiding in conceptualizing the time intervals that separate various land cover states recorded in the source cartographic materials.

Figure 2 depicts the appearance of individual maps listed in Table 1 covering the study area (A-H) at a reduced scale.

### Visualization and data processing

Our method of result visualization will be a cartographic presentation method, which results from the overlapping of areas. Distinct extents represent wetland areas. The preparation of this type of visualization is preceded by data acquisition through the retrogressive method.

The retrogressive method in landscape studies involves examining landscapes by retracing steps from a more recent time period back to progressively older periods^68–71^. In cartographic studies, the retrogressive method is an approach that involves the analysis and interpretation of historical maps, charts, or aerial photographs to understand changes in land cover and landscape over time^72^. This method allows cartographers and researchers to examine past land cover patterns and identify historical trends and transformations in the landscape^73,74^. While older cartographic materials may lack the precision of modern surveys, they provide crucial baseline information for assessing historical land cover patterns and natural features with the use of modern GIS software^75,76^.

The foundation for the retrogressive vectorization of forest, grassland, and wetland from paper maps is georeferencing their scanned raster form to the current coordinate system to achieve the lowest possible root-mean-square error (RMSE), which, in the case of topographic maps, is approximately 1–2 mm on a paper map^77^. Considering the georeferencing methods of old maps^75^, we applied first-degree affine transformation to the currently valid UTM (Universal Transverse Mercator) coordinate system for topographic maps, zone 33, EPSG: 32,633 (Table 1).

**Figure 3:**
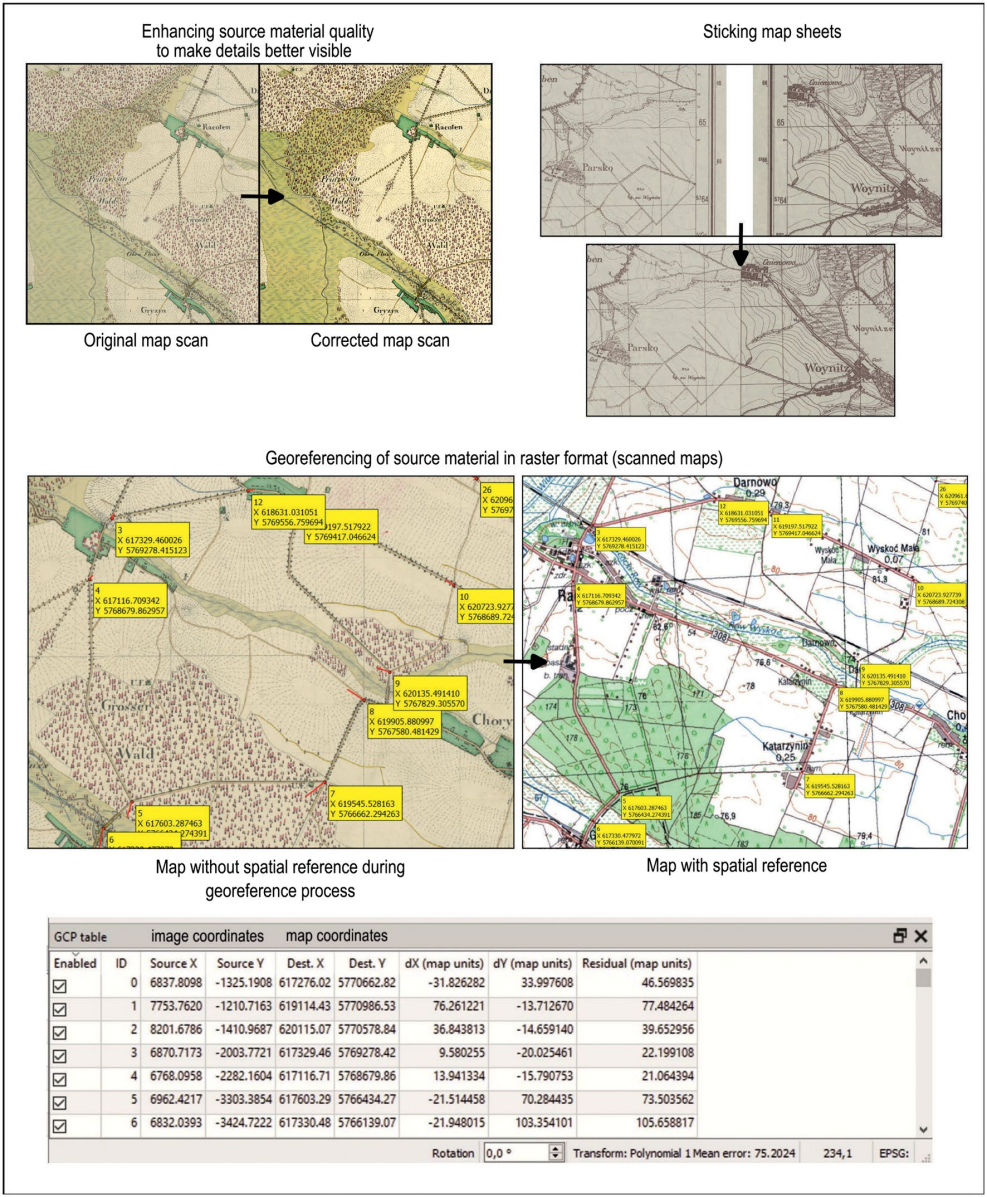
Stages of source data processing: Map color correction, map sheet integration, retrogressive georeferencing.

Figure 3 illustrates an example of the retrogressive georeferencing process for a map from 1826. It is visible on the left side, with control points overlaid that correspond to identical, identifiable points on the contemporary reference map with spatial context. In this case, the same road intersections were identified on both maps.

Based on the harmonization of the existing legends for the six newest maps from 1892 to 2023, the construction of the legend for the oldest map from 1793^48,49,51,67^ and supplementation with cartographic signs not appearing in the legend but visible on the map from 1826^78,79^, the following land cover categories were defined for research: forest, grassland, wetland, wet forest, wet grassland.

For vectorization, objects from the previous state were utilized to retain shapes where there were no changes in the defined land cover boundaries or to modify only a portion of an object's shape. In order to preserve topological rules during the vectorization process, appropriate settings in GIS software were enabled, including snapping options such as topological editing, snapping tolerance, or prevention of polygons overlapping (“avoid overlap”). The layers were automatically checked by GIS software to identify features that could potentially have invalid geometry. Any such errors were subsequently fixed.

## Results

**Figure 4:**
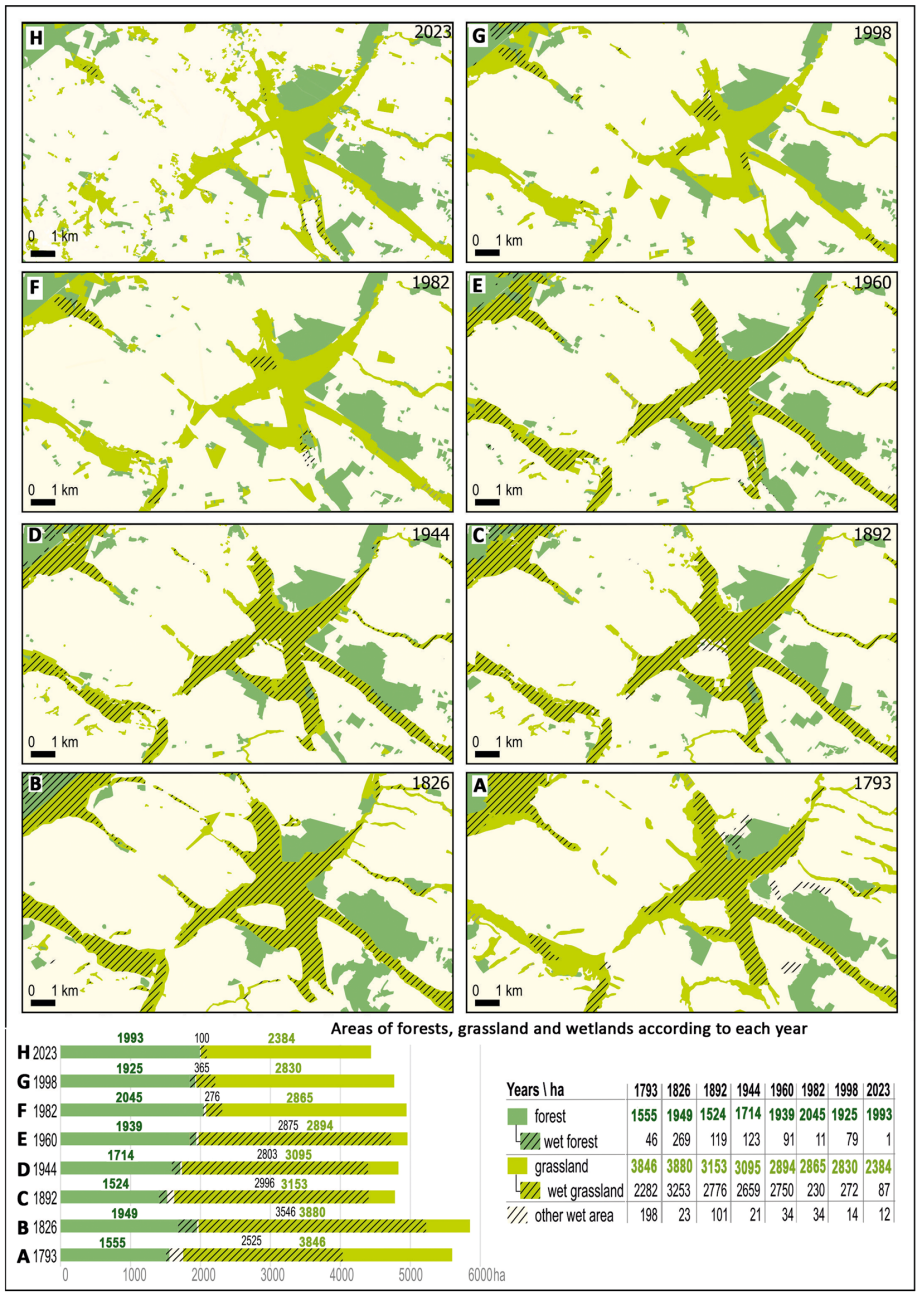
Areas of forests, grasslands and wetlands based on vectorization of topographic maps from 1793 to 2023. Below the maps, a bar chart illustrated the comparative distribution of different land cover types, including dry and wet grasslands, dry and wet forests, and other wetland areas.

The use of appropriate cartographic visualization allowed to reveal those spatial relationships that would not be visible on individual maps. For this reason, in Fig. 4, we can observe the elements of the landscape that have undergone spatio-temporal changes and those that have remained unchanged for 230 years, starting from 1793. Firstly, we can distinguish a large area of grasslands, which are located in the main line of canals and rivers. Secondly, we can distinguish large patches of forest in the vicinity of Kościan, which also in their core have not changed over this time. Both in the case of grasslands and in the case of forests, the core of the area has remained unchanged, while a certain envelope of these areas can be distinguished, which has fluctuated over the years.

### Long-term changes in forest cover

In 1793, the area of forests on Kościan Plain was 1555 ha. This forested area expanded to 1949 ha by 1826, but it decreased once more to 1524 ha in 1892. Over the subsequent years, the forested land gradually increased, reaching 2045 hectares by 1982. This peak acreage was slightly reduced, and as of 2023, the forested area stands at 1993 hectares. Throughout the period of 230 years, larger forested segments maintained or even expanded their acreage, with only a few smaller forested spots disappearing. Conversely, new areas were afforested.

Currently, in the structure of the Kościan Plain, there are nearly no forests growing on wetlands. However, in the past, such land cover were present to a slightly greater extent, especially in the year 1826 when the area of forests growing on wetlands reached as high as 14%.

### Long-term changes in grassland cover

In 1793, the area covered by grasslands was estimated as 3846 ha. The majority of this land was situated in wetland areas, (2282 ha), while non-wetland habitats overgrown by grasslands accounted for 1564 ha. The grassland area underwent substantial growth by 1826, expanding to 3880 ha. This expansion was primarily attributed to the significant increase in wetland grasslands, which encompassed 3253 ha, whereas grasslands in drier habitats decreased dropping to 627 ha.

Subsequently, by 1892, the grassland area was decreasing, both in wet and drier habitats and it was 2776 ha and 377 ha respectively. Over the next 50 years, land management remained relatively stable, with a slight increase in grasslands within drier habitats and a decline in wetlands. In 1960, a decrease in grassland coverage was observed, with wetland grasslands expanding to 2750 ha, while only 144 ha of grassland persisted outside of wetlands. Overall grassland coverage diminished by 201 ha, reaching 2894 ha.

By 1982, the total grassland area remained nearly constant (decreasing by only 29 ha). However, a significant decline in wetland grasslands (only 230 ha) was noted, with drier habitats taking their place (2626 ha). The situation exhibited stability through 1998, but by 2023, the overall grassland area had diminished by 2487 ha. This reduction included merely 87 ha of wetland grasslands, accompanied by a decreasing extent of other grasslands.

Over the past 230 years, grasslands have undergone profound changes in their surface area and habitat types, but the main grassland complexes have maintained their location along the watercourses. Nevertheless, a strongly advanced unfavorable process of fragmentation of grassland areas is visible. In recent years, new grasslands have been established in many drier areas, but these are mostly very small enclaves.

**Figure 5:**
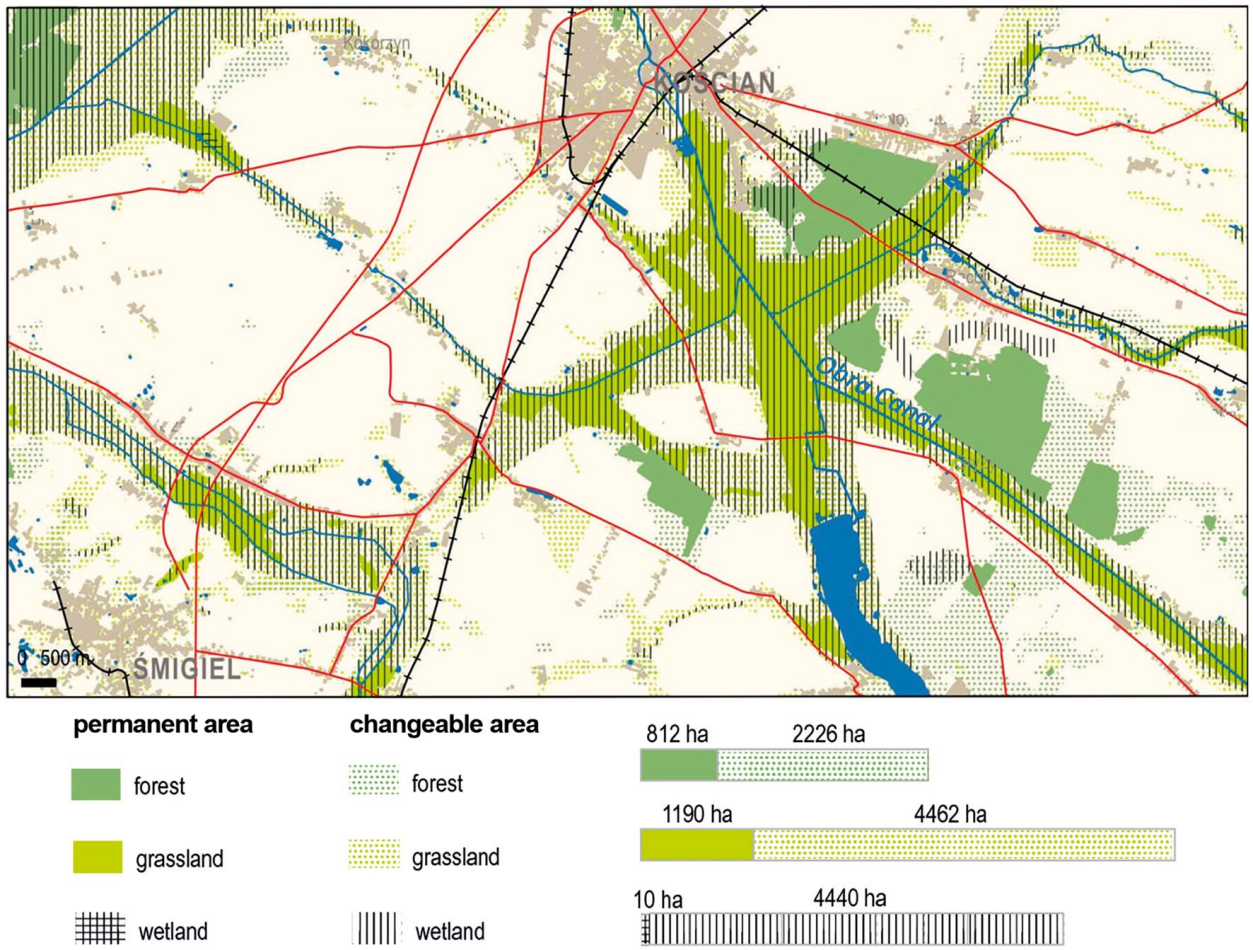
The map displays permanent areas and changes in forests, grasslands, and wetlands on the Kościan Plain between 1793 and 2023. It includes topographical features such as settlements, roads, railways, rivers, canals, and lakes.

The second of the developed cartographic visualizations serves as a synthesis of the results of landscape changes throughout the entire time analysis (Fig. 5).

### Long-term changes in wetland cover

In 1793, a significant portion of the landscape, covering 2525 hectares, was comprised of wetlands, with the majority of it being utilized as grasslands (90%), while the forested wetland area was marginal (2%). By 1826, the wetland area had notably expanded to 3546 hectares, maintaining its predominant use as grasslands (92%). Over the following century, the wetland area gradually decreased, and by 1960, it had dropped to 2875 hectares, with grasslands still dominating (96%).

In 1982, a drastic reduction in the wetland area was detected, leaving only 276 hectares remaining, consisting mainly of wetland grasslands (87%) and some wetland forest (12%). The wetland area experienced a slight increase, reaching 365 hectares in 1998. However, by 2023, only 100 hectares of wetlands remained.

## Discussion

The Kościan Plain remained relatively untouched by industry, with sustainable agricultural practices and limited urbanization due to a small human population in 1793. However, local timber harvesting for construction, fuel, and other needs may have been prevalent^55^.

The most significant changes were observed in the wetland area. Over 200 years, a progressive drying of habitats occurred. This slow process over 150 years was primarily driven by water regulation and land reclamation efforts initiated in the nineteenth century^37,65^. In 1982, a dramatic reduction in wetland area occurred, with only 276 hectares remaining, primarily consisting of wetland grasslands (87%) and some wetland forest (12%). The wetland area experienced a slight increase, reaching 365 hectares in 1998. However, by 2023, only 100 wetlands remained. This was primarily the result of large-scale land reclamation efforts initiated in the late 1960s and carried out during the 1970s to the early 1980s^62,64^.

On the one hand spatial arrangement of the landscape within the designated preservation areas in the Greater Poland Province reveals that these protected regions exhibit a notably rich and diverse array of natural elements, with a high density of different natural units^80^. On the other hand there was an increase in the share of agricultural areas by 73–81% and an increase in forest areas by 10–16.4% in the communes of Kościan and Śmigiel in the period between 1989 and 2005^39^. Therefore, both of these growths, which are also visible from the study results (with a 7.9% increase in forest cover), could have been influenced by the decrease in grasslands by 6.2%.

The transition towards afforestation, observed between 1944 and 1960, exemplifies a broader trend in land management practices across Europe^81^. Over this temporal span, a discernible trend emerges, indicating a transition towards afforestation of previously cleared lands^23^. This pattern reflects urbanization processes that may result in the abandonment of land, facilitating forest regrowth, a phenomenon observed in the Greater Poland Province^82^ or in the Carpathians^83^. The efforts to expand forest cover during this timeframe demonstrate a concerted endeavor to restore ecosystem services, enhance biodiversity, and mitigate the environmental impacts of human activities.

Grasslands have seen significant surface area changes and shifts in habitat types over the past 230 years. While the main grassland complexes have generally remained along watercourses, there is evidence of a notable unfavorable trend of fragmentation in grassland areas. Recent years have witnessed the establishment of new grasslands in drier areas, albeit in smaller enclaves. The decline and fragmentation of grasslands can be attributed, in part, to the intensification of agricultural production in Europe, as noted by Cousins^33^ and Kiviniemi and Eriksson^34^. This trend is not unique to the Kościan Plain but is a global phenomenon affecting various landscapes, including mountains, boreal regions, lowlands, and islands, as documented by Monteiro et al.^27^ and Aune et al.^84^.

The suggested method for studying the area using the topographic map sheet aimed to be universal, applicable to all handwritten topographic maps from the eighteenth century for the North European Plain^49^. The Map of Lower Saxony (Kurhannoversche Landesaufnahme 1764–1786) serves as an example of a handwritten topographic map for which the proposed method can be applied here due to the circumstances of the map's creation. The constant increase in the number of citizens in the Hanoverian Electorate had led to the necessity of expanding cultivable areas, including the drainage of wetlands, starting from the late seventeenth century. This need to cultivate moors and drain other extensive wastelands in Lower Saxony prompted efforts to create detailed maps^51^.

## Conclusions

Overall, the landscape changes on the Kościan Plain reflect a complex interplay between historical events, human activities, and natural processes. In conclusion, the existence of stable permanent areas in both grasslands and forests has contributed to the overall preservation of these landscapes on the Kościan Plain. The conservation of these permanent areas, along with the preservation of drainage canals, has played a vital role in maintaining a significant portion of permanent grasslands and forests.

This study successfully achieved its goal of quantifying changes in forest, grassland, and wetland areas and their distribution in the Kościan Plain, Poland, on the European Plain over the past 230 years. To achieve this objective, we utilized topographic maps as a reliable historical data source. Specifically, the study assessed changes in forest, grassland, and wetland areas, examining their durability over the study period. Wetlands have undergone significant reductions over time, with a drastic decrease in 1982. Land reclamation efforts and environmental factors have driven wetland loss. By 2023, only a fraction of wetlands remained.

Over the past two centuries, the grassland areas in the Kościan Plain fluctuated, influenced by wetland and drier habitat dynamics. Wetland grasslands expanded by 1826 but later declined. By 2023, overall grassland area had diminished remaining modified habitats still situated along watercourses exhibiting a concerning trend towards increased fragmentation. On the other hand the forested area has experienced limited fluctuations, also having a growth tendency peaking at in 1982. Forests on wetlands, which were always marginal, have nearly disappeared.

In this study, landscape metrics, as utilized in literature^53,85–87^, were not employed. Our methodology differs in its emphasis, focusing not on the indicative determination of the shape, fragmentation, or dispersion of land cover, but rather on identifying areas where land cover types have remained unchanged over the years (referred to as "permanent"). This approach also considers changeable areas of occurrence and incorporates graphical solutions for effective map representation.

In addition to the quantification of landscape changes, the study proposed a universal method for cartographic visualization. This method aims to support the effective visualization of changes in forest, grassland, and wetland areas in the North European Plain, providing valuable tools for future research and land-use planning efforts. The comprehensive analysis presented in this study provides valuable insights into the historical dynamics of the Kościan Plain's landscape and contributes to our understanding of the long-term impacts of human activities and urbanization on natural ecosystems. The proposed cartographic visualization method offers a practical tool for researchers and policymakers to monitor and manage landscape changes effectively.

## Data Availability

The datasets used and/or analyzed during the current study available from the corresponding author on reasonable request.
